# The Remapping of Time by Active Tool-Use

**DOI:** 10.1371/journal.pone.0146175

**Published:** 2015-12-30

**Authors:** Filomena Anelli, Michela Candini, Marinella Cappelletti, Massimiliano Oliveri, Francesca Frassinetti

**Affiliations:** 1 Department of Psychology, University of Bologna, Bologna, Italy; 2 Fondazione Salvatore Maugeri Hospital IRCCS, Castel Goffredo, Italy; 3 Institute of Cognitive Neuroscience, University College London, London, United Kingdom; 4 Department of Psychology, Goldsmiths College, University of London, London, United Kingdom; 5 Department of Psychology, University of Palermo, Palermo, Italy; 6 NeuroTeam Life and Science, Palermo, Italy; Ludwig-Maximilians-Universität München, GERMANY

## Abstract

Multiple, action-based space representations are each based on the extent to which action is possible toward a specific sector of space, such as near/reachable and far/unreachable. Studies on tool-use revealed how the boundaries between these representations are dynamic. Space is not only multidimensional and dynamic, but it is also known for interacting with other dimensions of magnitude, such as time. However, whether time operates on similar action-driven multiple representations and whether it can be modulated by tool-use is yet unknown. To address these issues, healthy participants performed a time bisection task in two spatial positions (near and far space) before and after an active tool-use training, which consisted of performing goal-directed actions holding a tool with their right hand (Experiment 1). Before training, perceived stimuli duration was influenced by their spatial position defined by action. Hence, a dissociation emerged between near/reachable and far/unreachable space. Strikingly, this dissociation disappeared after the active tool-use training since temporal stimuli were now perceived as nearer. The remapping was not found when a passive tool-training was executed (Experiment 2) or when the active tool-training was performed with participants’ left hand (Experiment 3). Moreover, no time remapping was observed following an equivalent active hand-training but without a tool (Experiment 4). Taken together, our findings reveal that time processing is based on action-driven multiple representations. The dynamic nature of these representations is demonstrated by the remapping of time, which is action- and effector-dependent.

## Introduction

In everyday life spatial and temporal information is constantly necessary for action: reaching, pointing, or grasping an object all need an accurate estimate of spatial and temporal features of the environment. An interesting aspect of space processing is the existence of multiple space representations depending on the extent to which an action can be performed on them [1,2,3]. In particular, near/reachable and far/unreachable spaces have been defined as the space inside and outside the arm’s reach distance [4].

Behaviourally, a dissociation between far and near space has been reported in healthy adults. During line bisection and landmark tasks, participants typically show a leftward bias (pseudoneglect) in near space that gradually shifts rightward as the stimulus line is moved further away [5,6,7].

Anatomically, animal studies supported the far/near space dissociation indicating that the frontal eye field and the lateral inferior parietal regions are the neural substrate for the far space representation, whereas the rostral part of the inferior parietal lobe and the ventral intra parietal regions support the near space representation [3]. In humans, several studies support this functional dichotomy showing that a more dorsal component, such as right parietal regions, is involved in near space processing, whereas a more ventral stream component, such as right ventral occipital areas, is involved in far space processing [8,9,10].

Interestingly, space representation is dynamic and the boundary between near and far space can be flexible; for instance, near space can be extended by performing goal-directed movements through the use of a tool, therefore making far space closer (for evidence in monkeys, see 4). In healthy adults, the dynamic properties of space representation have been supported by the observation that a tool-use training can impact on the representation of our own reaching space [2,11,12,13,14]. Moreover, the effects of tool-use support the functional distinction between ‘working’ space (i.e., the near space representation involved in goal-directed action) and ‘protective/defensive’ space (i.e., the near space representation involved in the protection of the body) [15]. According to this distinction, tool-use selectively alters the size of the working space.

Taken together, these data indicate that tool-use can influence the computation of space by extending near space to include the space accessible by a tool (far space). Thus, the definition of near and far space may depend upon action potentiality [16].

Space is not only multidimensional, but it is also known for interacting with other dimensions of magnitude, for instance time [17,18]. A growing literature often grouped within the ‘Conceptual Metaphor Theory’ documented the cross-domain link between space and time, positing that we understand abstract concepts (such as time) by mapping them onto more concrete ones (such as space) [19,20,21,22,23,24]. Indeed, it has been shown that we use a spatial map to represent time [25,26]. For example, in a duration judgment task of lateralized visual stimuli, healthy subjects overestimate durations in the right space and underestimate durations in the left space [27]. Moreover, judgments are faster for short temporal durations in the left space and long temporal durations in the right space [28], which suggests that time is encoded with an ascending left-to-right spatial order (Mental Time Line, MTL) (for a review, [18]).

Here, we considered the near/far dichotomy to study the interaction between space and time. More specifically, we investigated whether time can be influenced by a reachable/near and unreachable/far dimension of space, and whether time processing can be modulated by tool-use training. To address these questions, we tested young healthy participants in a time bisection task requiring to reproduce half of the duration of visual stimuli presented in near and far space. To adjust stimulus’ size for visual angle, a bigger stimulus was presented in far relative to near space. Thus, to exclude that a difference between near and far could be due to stimulus’ size rather than to a difference in space, small and big stimuli were presented in the two spaces. We reasoned that if near and far spaces are encoded on the basis of action potentiality, and if actions in space are not only based on spatial but also on temporal coordinates, then a different bias may be expected depending on the portion of space where the temporal stimulus is presented. In line with the literature on space perception [6,7], we predicted that a leftward bias in near space and a rightward bias in far space may be observed in our temporal task, for both small and big stimuli.

Secondly, we examined whether an active tool-training (i.e., a training during which participants performed goal-directed actions) may lead to time remapping, similar to what has been shown for space [2,11,12,13,14]. If time estimation can be modulated by action, then a time remapping in far space may be observed; thus stimuli duration in far and near space may be perceived as the same following tool-training.

## Experiment 1: Active Tool-Training in Far Space

### Materials and Methods

#### Participants

Twenty-two right-handed undergraduate students from the University of Bologna provided written consent to participate in the experiment (6 males, mean age±sd: 24±2.24 years, age range: 20–27). All participants had normal or corrected-to-normal vision, no history of neurological or psychiatric diseases. All were naive as to the purpose of the study and were debriefed about the experimental aims at the end of the experiment.

#### Ethics statement

All the experiments reported were carried out in accordance with the 2008 Helsinki Declaration and approved by the Ethics Committee of the University of Bologna.

#### Apparatus, stimuli, and procedure

Time perception was investigated with a time bisection task previously successfully used in healthy and neuropsychological patients [29,30,31,32]. Participants sat in front of a 15-inch color monitor, aligned with the subject's vertical midline at eye-level, with their dominant hand on the mouse. E-Prime 2.0 software was used for stimulus presentation and response collection.

**Fig 1:**
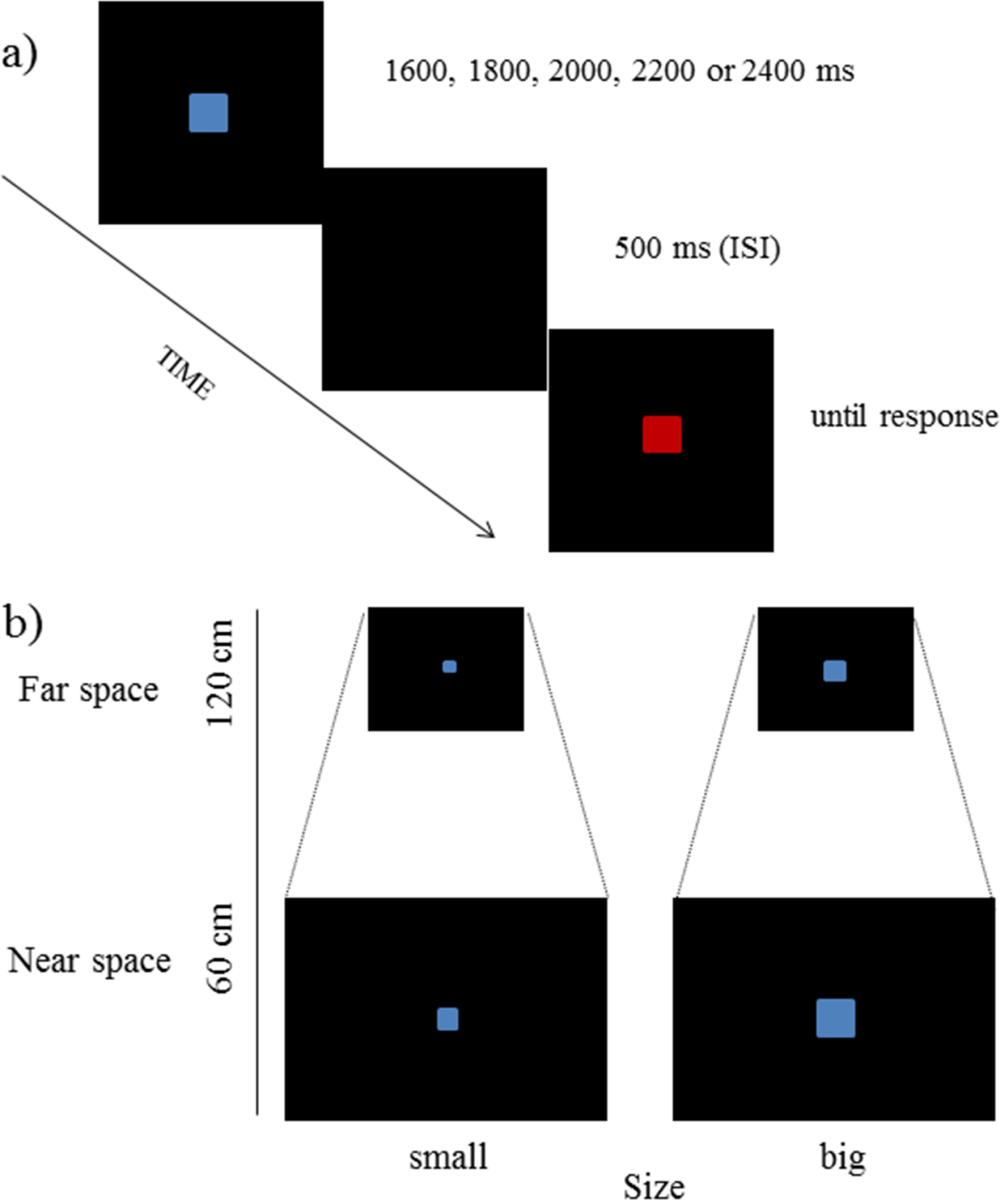
Stimuli used in the temporal bisection task. (a) Temporal structure of a representative trial. (b) Stimuli dimension depicted according to near and far space.

The experiment consisted of two phases. In the time encoding phase, a blue square was displayed on a black background for one of the following durations: 1600, 1800, 2000, 2200 or 2400 ms. In the time bisection phase, following a 500 ms-ISI a red square appeared on the monitor in the same position of the blue one; participants reproduced with a button press half of the duration of the blue square (Fig 1). No feedback was given. Inter-trial timing was fixed at 2000 ms.

The experiment consisted of three consecutive sessions, each separated by a 5-min interval. The whole experiment lasted about 50 minutes. During the first and third session, participants performed a time bisection task, whereas in the second session they performed a tool-use training.

During the first and third session, each participant performed the time bisection task in two spatial positions (near and far space) and repeated it twice, before (pre-training session) and after tool-use training (post-training session), for a total of four conditions. The order of the two spatial positions within the first session was counterbalanced across participants, and kept constant within participants during the post-training session.

In the Near space condition, participants seated at a distance of 60 cm from the monitor, in the Far space condition they were 120 cm away. Both distances refer to the eyes-to-screen distance and were measured by the experimenter through a metal tape measure. The computer monitor was moved at two different distances along a table, in order to maintain the same position for both screen and plane of response. Response device was kept in the same position in both spaces. Stimuli were squares centrally displayed on the monitor. Stimulus’ size was adjusted for visual angle. To exclude spurius effects due to different stimulus sizes presented in near and far space, two sizes were used: small square stimuli, corresponding to 1cm x 1cm in Near space (0.95 degrees of visual angle), and 2cm x 2cm in Far space (0.95 degrees of visual angle). Big square stimuli were 2cm x 2cm in Near space (1.91 degrees of visual angle), and 4cm x 4cm in Far space (1.91 degrees of visual angle). Small and big square stimuli were randomly presented.

For each testing session (pre- and post-training) there were 120 randomly presented trials, 60 in near and 60 in far space (12 trials for each of the 5 intervals), for a total of 240 trials. Before each pre-training session, subjects performed a practice session, with twenty 2000 ms-square, where the stimulus sizes were randomized. Participants were instructed to maintain a stationary position, to fixate the screen central point during the task, and to respond with their right hand.

Between the first and third session, participants sat at the table and performed a 15-minutes active tool-use training. This consisted of reaching small objects (i.e., fiches and caps of different colors) placed on the table in far space, at a distance of 120 cm from the participant, holding a rake (tool) with their right hand, namely the same hand used to execute the time bisection task. Small objects were randomly presented in the participants’ midsagittal axis (0°), or at 10° and 20° to the left and to the right of the central position (-10°, -20°, +10°, +20°). Each participant performed about a total of 90 reaching movements in order to bring the objects close to the body with no time constraint. The tool used during the training was a 70 cm long plastic rake, composed of an ergonomic handle, a shaft, and a final part consisting of seven teeth (each 5 cm height) useful to grasp and bring objects, with a total weight of 120 grams.

### Results and Discussion

The means of reproduced temporal durations (ms) were entered into a repeated-measures ANOVA with *Session* (pre- and post-training), *Space* (near and far), *Dimension* (small and big), and *Interval* (1600, 1800, 2000, 2200, and 2400 ms) as within-subjects factors. Post-hoc tests Newman-Keuls corrected for multiple comparisons were performed on significant effects and the magnitude of effect size was expressed by η^2^_p_.

**Fig 2:**
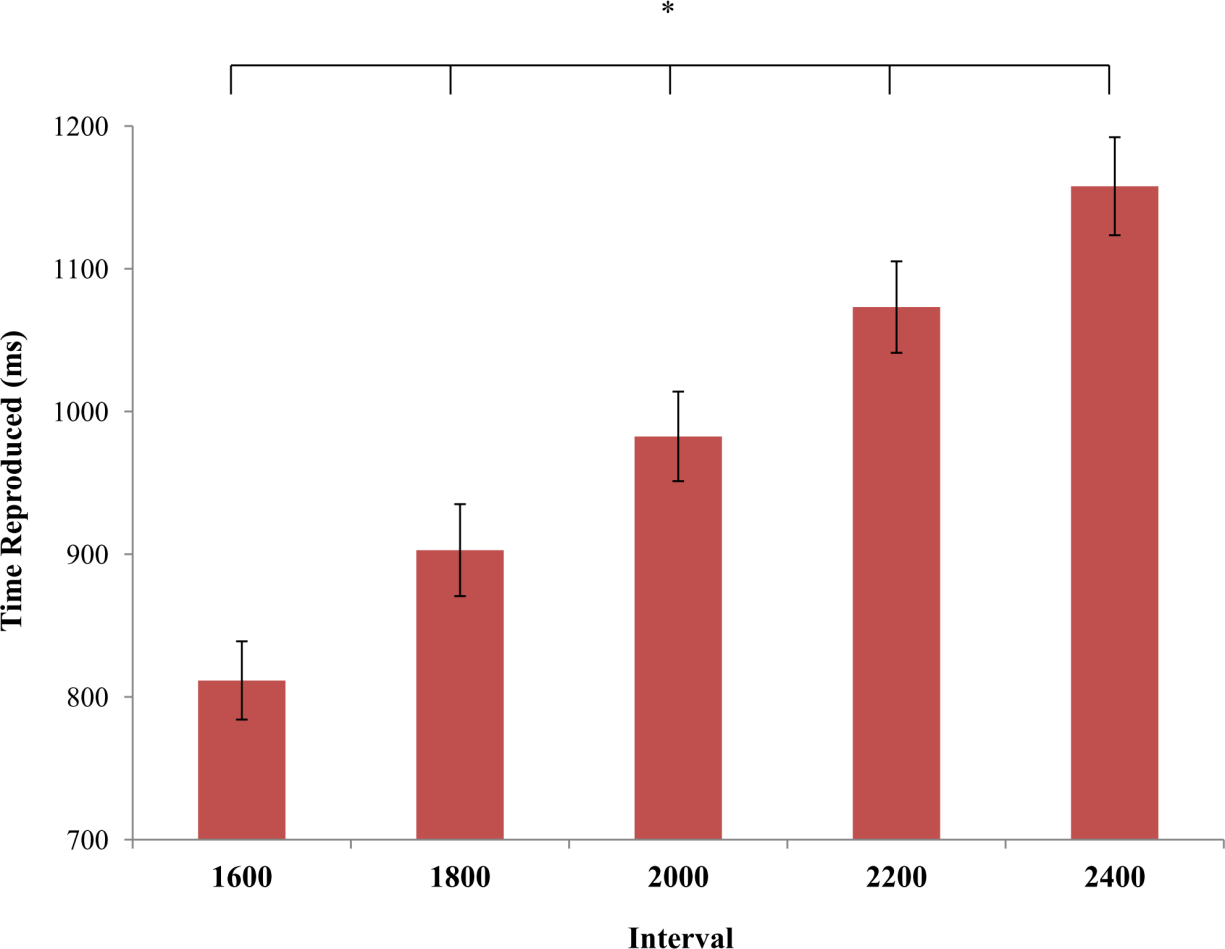
*Interval* effect. Values are in ms and error bars depicted SEM. Asterisks indicate significant differences (p < .05).

**Fig 3:**
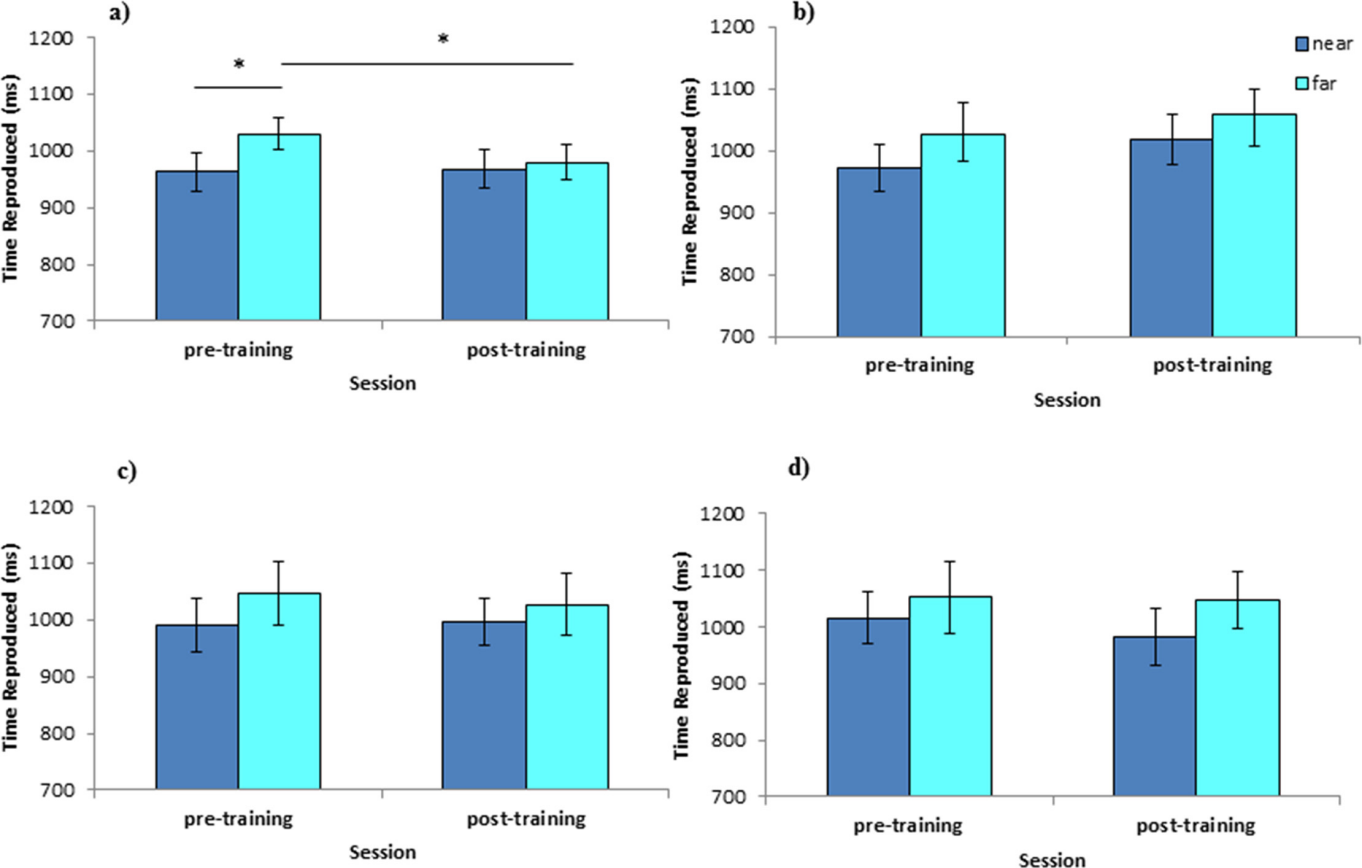
*Space* x *Session* interaction in the four experiments (Experiments 1–4 in panels a-d respectively). Values are in ms and error bars depicted SEM. Asterisks indicate significant differences (p < .05). Note that *Space* x *Session* interaction is not significant in Experiments 2–4, thus asterisks are not reported.

The main effect of *Space* was significant [F(1,21) = 15.56, η^2^_p_ = .43, p < .001], because participants showed a rightward deviation when they bisected temporal intervals in far (1005 ms) compared with near space (966 ms). *Interval* was also significant [F(4,84) = 112.47, η^2^_p_ = .84, p < .001], because all intervals differed significantly from each other (veridical durations: 1600, 1800, 2000, 2200, 2400 ms; estimated half durations: 811, 903, 982, 1073, 1158 ms, respectively; post-hoc tests p < .001 for all comparisons), (Fig 2). Importantly, the interaction between *Session* and *Space* was also significant [F(1,21) = 9.76, η^2^_p_ = .32, p < .01], because in the pre-training session participants showed a significant leftward bias when they bisected temporal intervals in near (964 ms) compared to far condition (1031 ms, p < .001). By contrast, no difference between near and far condition was observed in temporal bisection after tool-training (968 vs. 980 ms, respectively, p = .35). Crucially to our aim, when pre- and post-training sessions were directly compared, far conditions significantly differed from each other (p < .001), whereas near conditions did not (p = .75). However, no significant differences emerged between near space in the pre-training session and far space in the post-training session (p = .42), (Fig 3A). There were no other significant effects or interactions (all ps>.11).

Experiment 1 shows that a specific bias in temporal processing depends on the spatial location where the temporal stimulus was presented. In particular, we found a leftward bias in near space and a rightward bias in far space, a dissociation that supports the existence of action-driven multiple time representations.

Second, we report a remapping of time following an active tool-use training. Indeed, after tool-training, temporal stimuli presented in far space were processed as if those occurred in near space. This effect suggests that time perception can be modulated by an active tool-use which depended on whether an action was possible in space. Thus stimuli in far space, which was previously unreachable, are now reachable in near space. Our results complement previous data on tool-use effect on space remapping (e.g., [2,4]), demonstrating that time remapping can occour following an active tool-use which extends action potentiality.

Although the tool allowed participants to reach and retrieve objects placed in a far space (i.e., at a distance of 120 cm from participants), it is yet unclear whether the mere passive holding of a tool (i.e., a passive tool-training), rather than the action performed, may have induced time remapping. To address this issue, we conducted a control study (Experiment 2) requiring participants to perform a passive tool-training instead of an active one. We reasoned that if, as we advanced, time remapping is action-dependent, then a time remapping in far space should not be observed following a passive tool-training.

## Experiment 2: Passive Tool-Training in Far Space

### Materials and Methods

#### Participants

Fifteen new right-handed young adults (4 males, mean age±sd: 24±2.62 years, age range: 20–29) from the same pool as in Experiment 1 were recruited.

#### Apparatus, stimuli, and procedure

The apparatus, stimuli, and procedure were the same as outlined in Experiment 1, except for the following differences. First, only small stimuli were used corresponding to 1cm x 1cm in Near space (0.95 degrees of visual angle) and 2cm x 2cm in Far space (0.95 degrees of visual angle). This is because in Experiment 1 no difference was found between the two different stimulus sizes (i.e., small and big). Thus, for each testing session (pre- and post-training) there were 60 randomly presented trials, 30 in near and 30 in far space (12 trials for each of the 5 intervals), for a total of 120 trials.

Second, the training performed between the two experimental sessions was also different. Participants sat at the table and held the same tool used in Experiment 1 with their right hand for the whole training session (15-minutes). The experimenter randomly placed small objects (i.e., fiches and caps of different colors) on the table near the tip of the tool at the same locations as in the Experiment 1 (i.e., at a distance of 120 cm). Participants carefully observed the objects’ position but did not perform any movement to reach the objects. In order to maintain a constant level of attention during the training and to ensure that their attention was directed toward the tip of the tool throughout the whole training, participants were occasionally asked to verbally report the color and the spatial position of the object presented. Hence, participants performed a training which did not require the active use of the tool.

### Results and Discussion

The means of reproduced temporal durations (ms) were entered into a repeated-measures ANOVA with *Session* (pre- and post-training), *Space* (near and far), and *Interval* (1600, 1800, 2000, 2200, and 2400 ms) as within-subjects factors. Post-hoc tests Newman-Keuls corrected for multiple comparisons were performed on significant effects and the magnitude of effect size was expressed by η^2^_p_.

The main effect of *Space* was significant [F(1,14) = 10.38, η^2^_p_ = .43, p < .01], because there was a rightward deviation in bisecting temporal intervals in far (1042 ms) compared with near space (996 ms). Interval was also significant [F(4,56) = 87.06, η^2^_p_ = .86, p < .001], because all intervals differed significantly from each other (veridical durations: 1600, 1800, 2000, 2200, 2400 ms; estimated half durations: 846, 943, 1044, 1104, 1156 ms, respectively; post-hoc tests p < .01 for all comparisons). *Session* (η^2^_p_ = .18, p = .09) and, crucially, its interaction with *Space* (η^2^_p_ = .04, p = .49) were not significant (Fig 3B).

Experiment 2 showed a leftward bias in near space and a rightward bias in far space, thus replicating the near/far dissociation in temporal processing found in Experiment 1. Crucially, no remappig of time was found following a passive tool-training, such that the near/far dissociation was still present after tool-training. This evidence clearly shows that time remapping is action-dependent and does not occur following a passive tool-use, namely while holding a tool without performing an action.

Although data from Experiments 1 and 2 clearly demonstrate the action-dependence of the tool-use effect, it may yet be possible that time remapping occurs regardless of the specific effector used during the training. To address this question, we conducted another control study (Experiment 3) requiring participants to perform an active tool-training. Using the same procedure described in Experiment 1, participants held the tool with the left hand, namely the opposed hand to that used to execute the time bisection task, instead of the right hand, as they did in Experiment 1. Based on the view that tool-training specifically induces a transient expansion of the space around the used effector [4,33,34], we therefore predicted that time remapping may be effector-dependent, and that no effect may be observed after a training with the left hand.

## Experiment 3: Active Tool-Training in Far Space with Left Hand

### Materials and Methods

#### Participants

Fifteen new right-handed young adults (5 males, mean age±sd: 23±3.85 years, age range: 18–30) from the same pool as in Experiment 1 were recruited.

#### Apparatus, stimuli, and procedure

The apparatus, stimuli, and procedure were the same as outlined in Experiment 2, the only difference being the training. Participants sat at the table and performed the same 15-minutes active tool-use training described in Esperiment 1, but they were required to use the tool with their left instead of the right hand. Hence, while participants were instructed to respond with their right hand during the temporal bisection task, they used the left hand during the active tool-use training.

### Results and Discussion

Here we performed the same analysis described in Experiment 2. The main effect of *Space* was significant [F(1,14) = 5.36, η^2^_p_ = .28, p < .05], because participants showed a rightward deviation when they bisected temporal intervals in far (1037 ms) compared with near space (994 ms). *Interval* was also significant [F(4,56) = 63.54, η^2^_p_ = .82, p < .001], because all intervals differed significantly from each other (veridical durations: 1600, 1800, 2000, 2200, 2400 ms; estimated half durations: 832, 930, 1033, 1108, 1175 ms, respectively; post-hoc tests p < .01 for all comparisons). *Session* (η^2^_p_ = .00, p = .82) and, crucially, its interaction with *Space* (η^2^_p_ = .03, p = .53) were not significant (Fig 3C).

These results replicated the dissociation found in Experiments 1 and 2 between time processing in near and far space. Moreover, this dissociation was present both pre- and post-training showing that an active tool-use training is not sufficient to induce a remapping of time if executed with a different effector from that used to perform the temporal task (i.e., left vs. right hand). This therefore suggests that tool-use effects on time processing are effector-dependent.

Although so far we demonstrated both the action-dependence and the effector-dependence of the tool-use effect for time remapping, it may be possible that this remapping occurs following any action, regardless of the instrument or effector used. To test this possibility, a final control study (Experiment 4) was carried out, whereby participants interacted with objects presented in near space using their right hand instead of the tool. We reasoned that if time remapping is strictly tool-based, then there should be no time remapping in far space following the hand-training.

## Experiment 4: Active Hand-Training in Near Space

### Materials and Methods

#### Participants

Fifteen new right-handed young adults (4 males, mean age±sd: 25±1.88 years, age range: 22–29) from the same pool as in Experiment 1 were recruited.

#### Apparatus, stimuli, and procedure

The apparatus, stimuli, and procedure were the same as outlined in Experiment 2, the only difference being the training. Participants sat at the table and performed a 15-minutes active training which consisted of reaching small objects using their own right hand instead of the tool, for the whole training session. The experimenter placed the same small objects used in previous experiments on the table, at a distance of 60 cm from the participants in the near space. Participants were asked to reach the objects and to bring them close to their body with a single hand movement. Hence, participants performed a training in the near space which did not require the use of the tool.

### Results and Discussion

Here we performed the same analysis reported in Experiments 2 and 3. The main effect of *Space* was significant [F(1,14) = 5.97, η^2^_p_ = .30, p < .05], because participants showed a rightward deviation when they bisected temporal intervals in far (1049 ms) compared with near space (998 ms). *Interval* was also significant [F(4,56) = 57.91, η^2^_p_ = .81, p < .001], because all intervals differed significantly from each other (veridical durations: 1600, 1800, 2000, 2200, 2400 ms; estimated half durations: 841, 938, 1037, 1124, 1181 ms, respectively; post-hoc tests p < .05 for all comparisons). *Session* (η^2^_p_ = .02, p = .56) and, crucially, its interaction with *Space* (η^2^_p_ = .04, p = .47) were not significant (Fig 3D).

These results replicated the dissociation between time processing in near and far space shown in Experiments 1, 2, and 3. This dissociation was present both pre-and post-training showing that merely performing actions with a hand rather than a tool was not sufficient to induce a remapping of time.

## General Discussion

This study explored whether time processing mirrors the multi-dimensional organization of space, and whether it can be equally modulated by a training, actively or passively executed with a tool or actively executed with hand. The existence of multiple representations of space is now well established [1,35], as well as the effects of tool-use on the boundaries between different spatial representations, which reflects the flexibility of space reachability [4]. Space is also known for interacting with time processing, suggesting a robust link between these dimensions [18], but whether time is also multi-dimensional and can be modulated by tool-use is not yet known.

Using an established time bisection paradigm [29,30,31,32], we first investigated whether time may be influenced by action potentiality in space. We found a dissociation between time in near/reachable space and far/unreachable space, indicating that multiple time representations exist. This is in line with multiple space representations revealed in performing line bisection and landmark tasks [1,6,7,35], whereby healthy adults show a left-to-right shift with increasing distance [6].

To our knowledge, one study has so far investigated time processing of imaginary objects located in either near/far representational space [36]. Participants were required to estimate the duration of an imagined analog clockface in two conditions. In near condition the clock had to fill the full imagery field; in the far condition the clock had to be imagined from a distance of about 6 metres. The duration estimation was shorter in far than in near space. However, since in the far condition the imagined stimulus was veridically smaller than in the near condition, this leaves open the possibility that participants may have processed the relationship between size and time, rather than space and time. Instead our results cannot be explained by an effect of stimulus’ size because we have adjusted the stimuli dimension in the near and far space. This manipulation, as well as the lack of a significant *Dimension* effect in the analyses, makes it less likely that our dissociation of near/far time could simply be explained by different stimuli size presented in near and far space.

Next, we examined whether an active tool-training compared to a passive tool-training and to an active hand-training may lead to a remapping of temporal duration, similar to what observed in the spatial domain [1,37]. We found that only following an active tool-use training (Experiment 1), participants were able to remap time in far space similar to what already found in space, whereby training typically makes stimuli in far space reachable in near space. Indeed, following an active tool-training temporal judgments did not differ in far and near space. Conversely, following both a passive tool-training (Experiment 2) and an active hand-training (Experiment 4) a remapping of time in far space did not occur. Remarkably, remapping was not observed even when a different hand was used during the active tool-use training and the temporal bisection task (i.e., left and right hand, respectively; Experiment 3).

These results suggest that tool-use training modifies and spatially extends our action potentiality, leading to a different coding of time on the basis of action potentiality in space. Indeed, time remapping proved to be action-dependent, since the extension of action potentiality specifically occured following the active tool-use rather than simply holding a tool. At the notion that tool-use modifies the working space [15], here we also highlight the temporal component of space. Recent evidence provided further support to the link between time perception and action potentiality. For instance, temporal judgments are more accurate and precise for visual stimuli moving accordingly to the human motor repertoire rather than non-biological kinematics, suggesting that observer’s motor knowledge implicitly affects time perception [38].

Different reasons may explain the effect of tool-training. One is in terms of a general attentional mechanism, such that using a tool may lead to a shift of spatial attention from the effector towards the side of space where the tip of the tool is located, that is the space made reachable during the tool-training (e.g., [39,40]; for a review, [41]). In this case, the tool training may enhance the salience of visual stimuli presented in the far space, a possibility that was excluded in Experiment 2 whereby participants passively held the tool and reported some information about the objects presented at the tip of the tool. This requirement may have induced participants to shift their attention toward far space during the passive tool-training in order to correctly report the required information, as also shown in other recent studies [42,43]. Here, passive tool-use training resulted in no remapping, therefore excluding that general attentional mechanism fully explained time remapping.

A second interpretation of the tool-based time remapping has to be considered, assuming a change in the *perception* of external visual space, based on the action-specific perception view. This claims that perception derives from the relation between the external environment and the ability to act within it [44]. Indeed, spatial targets are perceived closer than veridical when they are within reach of a tool rather than the hands, suggesting that space perception is influenced by intended actions ([14,45]; but see [46]). This alteration of perceived distance emerged only when coupled with the perceiver’s intention to act, and it is not observed when participants passively hold a tool [4,47]. Our results only partially support this interpretation since we found that time remapping is strictly dependent on the effector trained, and it does not emerge when participants used a different hand to perform the tool-use training and the temporal bisection task (Experiment 3).

A final explanation of the tool-use effect is in terms of a change in the Body Schema, namely the body representation used to plan and to execute an action. This suggests that following training, a temporary tool-embodiment would occur, such that the tool becomes part of the body (e.g., [1,12,13]; for a review, [34]). This is supported by the observation of changes in the kinematic parameters of arm movements after tool-training [2,11]. These kinematics changes would reflect somatosensory modifications in the Body Schema, as if participants performed the movements with a longer arm after tool-use. Similarly, tool-use induced a shift in the perceived midpoint of participants’ own forearm distally with respect to the pre-tool midpoint, in line with an increased internal representation of the arm length [42]. Hence, tool-embodiment affects not only the kinematic of arm movements, but also sensory body representation [2,11,42,43,48,49,50,51]. Our results are fully compatible with the tool-embodiment hypothesis since it accounts for the action-dependence of the time remapping effect (Experiments 1 and 2), as well as its effector-dependence (Experiment 3). Indeed, an increased internal representation of the arm length which was specific for the arm trained, explains why no time remapping was observed when tool-training and temporal bisection were executed with two different hands.

In conclusion, the current study demonstrates that action influences time perception, resembling the effect that action has on space and providing the first evidence for the flexible nature of time representation, whose boundaries can be dynamically modulated by active tool-use. Given the well-known effect of tool in the spatial domain, it is difficult to disambiguate whether tool-use changes time processing by modifying spatial features, or whether tool-use directly impacts on time processing without the mediation of space. We speculate that at least in our paradigm, the observed remapping may act on space and time, since these representations are mutually related and both coordinates contributed to action [29,52].
